# 

**DOI:** 10.1192/bjb.2021.63

**Published:** 2022-06

**Authors:** Mohammed Ahmed Rashid

**Affiliations:** MBChB, MSc, MRCGP, is a Clinical Associate Professor in the Faculty of Medical Sciences at UCL Medical School, London, UK. Email: ahmed.rashid@ucl.ac.uk

*Nice White Parents* is a podcast series about an ordinary middle school in New York.^1^ It charts how, over the school's 50-year history, White parents have consistently, and almost always unwittingly, exercised their enormous unsaid power in the public education system to directly and indirectly hamper the school's mission of providing a progressive and integrated education to children of all racial and socioeconomic backgrounds.

One of the most striking messages from the series is how White parents have been able to game all aspects of the education system and hoard resources for their own children. The White parents covered in the series had more time, influence and resources, and were therefore much better equipped to navigate the system and advocate for their children than Black and minority ethnic parents in the same city. Although the White parents were making seemingly justifiable and rational decisions, the collective impact of their decisions has been to perpetuate long-standing racial inequalities that go back several decades.

Listening to this podcast series, it was hard not to draw parallels with what I see as a clinician in the UK's National Health Service (NHS). Some scenarios that I encounter regularly bear an uncanny resemblance to it, such as parents ferociously seeking referrals to multiple agencies and specialists to get diagnoses for their children by lobbying multiple doctors and seeking second opinions. Other scenarios are less directly analogous, but nonetheless relate to power dynamics and individuals’ ability to navigate a complex system – wealthy professionals seeking NHS referrals on the basis of private medical reports, and individuals demanding specific investigations or treatments on the basis of informal conversations with medical family members or friends.

The nature of a publicly funded system, whether it is health or education or any other sector, is that every decision needs to be seen through two lenses – at the individual and the population levels. The traditional medical paradigm, and more specifically recent movements such as patient-centred care, encourage us to favour the individual lens. The danger of this, as we are seeing very powerfully in 2021 with the COVID-19 pandemic, is that it ignores the vulnerable, the less powerful and the voices that cannot shout as loud. Resources are inevitably finite and so each decision we take about one individual or one family can have very real and profound repercussions for many others.

Of course, not all White patients are manipulative, and not all manipulation is by White patients. But as the podcast series points out, nobody talks about White parents, and likewise in medicine, we rarely talk about White patients. Just like the ‘nice White parents’ in New York, my White patients are not acting maliciously and they are not fundamentally against the NHS values of compassion and equality. The problem is rather that they are rarely cognisant of their power and, more importantly, the system simply works better for those who can game it.

As we seek to achieve greater equity in healthcare, is it time we talked more about White patients?
